# Well-Tempered Metadynamics Simulations Predict the Structural and Dynamic Properties of a Chiral 24-Atom Macrocycle in Solution

**DOI:** 10.1021/acsomega.2c03536

**Published:** 2022-08-08

**Authors:** Riccardo Capelli, Alexander J. Menke, Hongjun Pan, Benjamin G. Janesko, Eric E. Simanek, Giovanni M. Pavan

**Affiliations:** † Department of Applied Science and Technology, Politecnico di Torino, 10129 Torino, Italy; ‡ Department of Chemistry & Biochemistry, Texas Christian University, Fort Worth, Texas 76129, United States; § Department of Chemistry, University of North Texas, Denton, Texas 76129, United States; ∥ Department of Innovative Technologies, University of Applied Sciences and Arts of Southern Switzerland, Polo Universitario Lugano, 6962 Lugano-Viganello, Switzerland

## Abstract

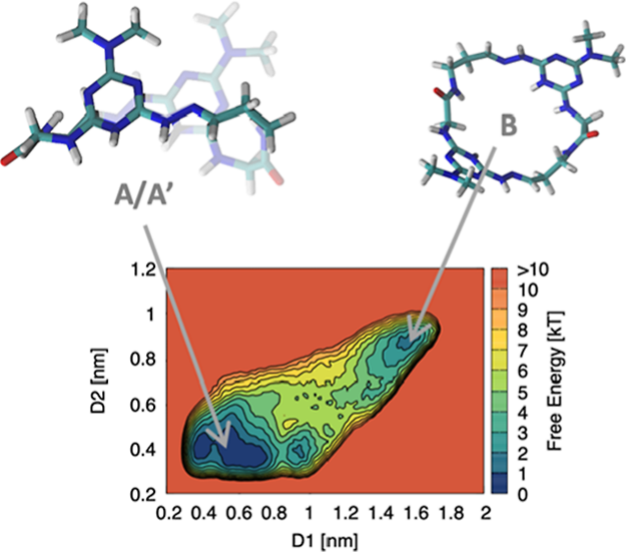

Inspired by therapeutic potential, the molecular engineering of macrocycles is garnering increased interest. Exercising control with design, however, is challenging due to the dynamic behavior that these molecules must demonstrate in order to be bioactive. Herein, the value of metadynamics simulations is demonstrated: the free-energy surfaces calculated reveal folded and flattened accessible conformations of a 24-atom macrocycle separated by barriers of ∼6 kT under experimentally relevant conditions. Simulations reveal that the dominant conformer is folded—an observation consistent with a solid-state structure determined by X-ray crystallography and a network of rOes established by ^1^H NMR. Simulations suggest that the macrocycle exists as a rapidly interconverting pair of enantiomeric, folded structures. Experimentally, ^1^H NMR shows a single species at room temperature. However, at lower temperature, the interconversion rate between these enantiomers becomes markedly slower, resulting in the decoalescence of enantiotopic methylene protons into diastereotopic, distinguishable resonances due to the persistence of conformational chirality. The emergence of conformational chirality provides critical experimental support for the simulations, revealing the dynamic nature of the scaffold—a trait deemed critical for oral bioactivity.

## Introduction

Current interest in macrocycles derives in great part from their potential application in medicine.^1−5^ Unlike small-molecule drugs that traditionally bind to well-defined active or allosteric sites, macrocycles can also access an alternate therapeutic paradigm by targeting protein–protein interfaces which are often disordered.^6^ Recognition of conformationally fluid regions on proteins can occur if a macrocycle adopts an extended conformation to display the requisite array of functional groups necessary for productive binding. However, this strategy presents a challenge. That is, an array of polar interactions can preclude transport across membranes and/or oral bioavailability.^7,8^ Accordingly, a dynamic “chameleon-like” character is desired wherein the macrocycle can adopt extended conformations that facilitate molecular recognition and compact structures that enhance lipophilicity to promote cellular access.^9,10^

Macrocycle **G–G** represents the simplest, most flexible macrocyclic scaffold of a panoply of potential compounds which can be accessed by functionalization of any of the 12 sites on this scaffold including the choices of (1) the auxiliary group (here, dimethylamine), (2) *N*-alkylation state of the hydrazine (here, H), (3) length of the acetal (here, three carbons), choice of the amino acid (here, G), and its *N*-alkylation state (here, H). Indeed, the opportunity for structural diversity on this scaffold that sits—based on its size and composition—on the border of molecules that are expected to follow Lipinski’s rules of 5 (Ro5) and those that are beyond them (BRo5) makes it a compelling choice for us to study.^8−10^ The glycine macrocycle, **G–G**, derives from acid-catalyzed dimerization of a simple monomer comprising a central triazine and auxiliary group (dimethylamine), a protected hydrazine group, and an acetal pendant on the glycine linker. It is available rapidly in three synthetic steps requiring two chromatographic purifications.

Predicting the conformational flexibility of a macrocycle prior to synthesis can greatly impact molecular design.^11,12^ Molecular simulations are a useful tool to this end.

**Chart 1:**
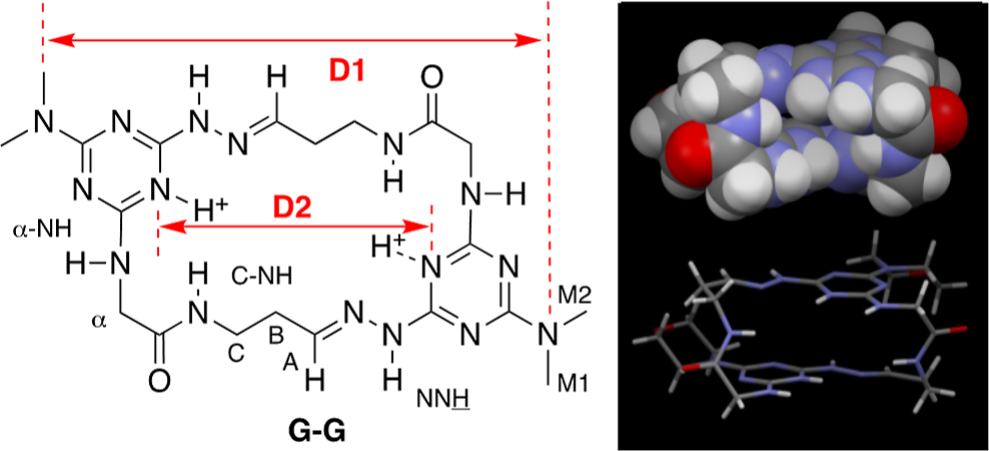
**G–G** Macrocycle, D1 and D2 WT-MetaD Collective Variables (CVs), and Labels Used in the NMR Spectra. The Solid-State Structure of a Morpholine Derivative Is Shown with the Solvent and Counterions Omitted for Clarity

While classical molecular dynamics (MD) simulations allow the exploration of configurations that differ by ∼2 kT in free energy, enhanced sampling approaches such as metadynamics are ideally suited for this task^13,14^ by preventing entrapment into local free energy minima. Metadynamics allows for an exhaustive exploration of the conformational landscape of complex macromolecules.^15^ Here, we use well-tempered metadynamics (WT-MetaD)^16^ simulations to characterize the conformational landscape of the 24-atom macrocycle referred to as **G–G** (Chart 1) in the explicit solvent and under experimentally relevant conditions.

## Results and Discussion

**Figure 1:**
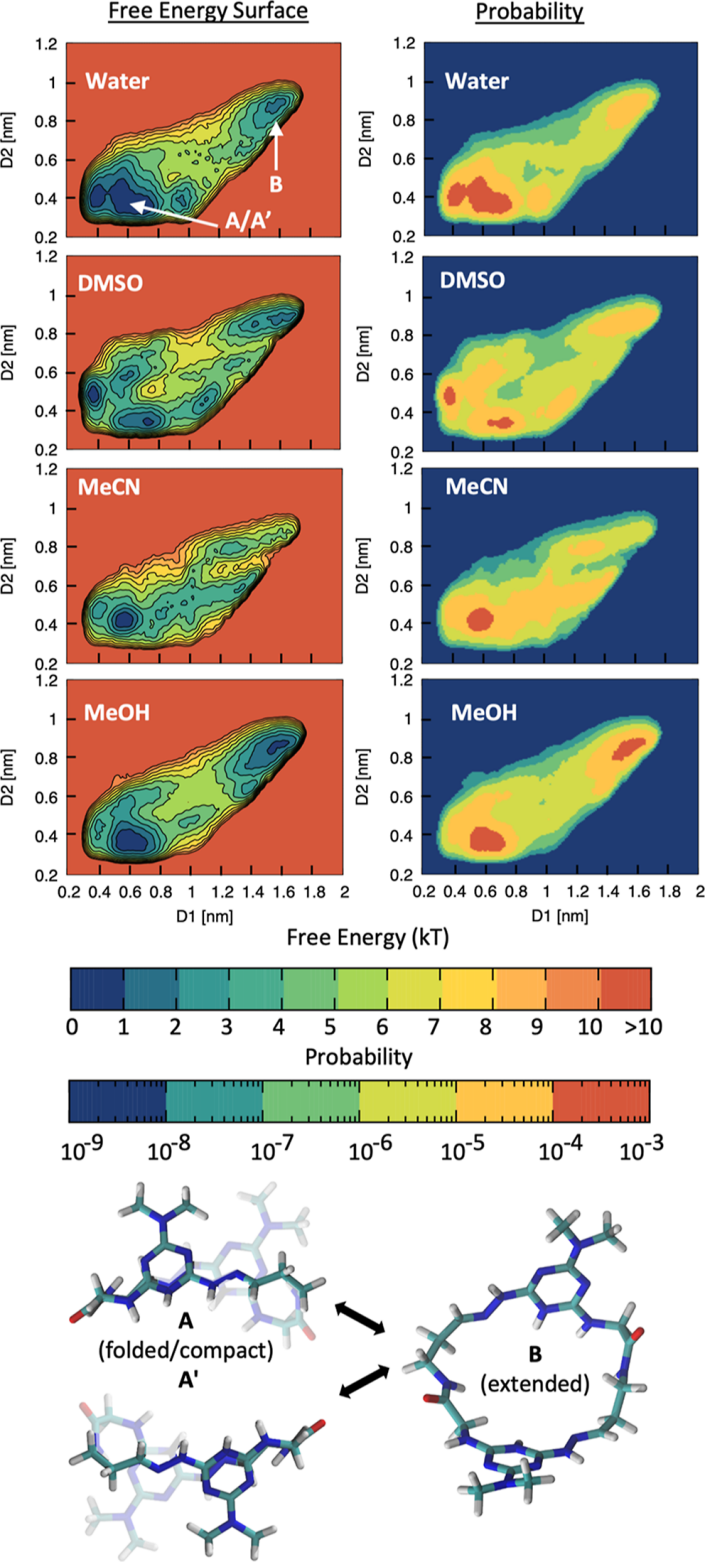
WT-MetaD simulations reveal two dominant local minima. Left: FESs obtained from WT-MetaD simulations of the **G–G** macrocycle in different explicit solvents as a function of the D1 and D2 distances (WT-MetaD CVs: *x*- and *y*-axes in the FESs). For all the FESs shown, the average error, evaluated by block averaging, is equal to 0.2 kcal/mol. Right: population probabilities for the **G–G** conformations on the D1–D2 plane computed from the FESs (eq 1). The data identify two most probable conformations for **G–G**, corresponding to free energy minima in the FESs: a compact folded structure (**A** and its enantiomer **A′**), in the lower-right quadrants, and a fully extended structure (B), in the upper-left quadrant. Bottom: representative snapshots of the dominant **A/A′** and **B** conformers. The arrows connecting them indicate the pathway of dynamic interconversion **A** ⇔ **B** ⇔ **A′** (and back) observed at low temperatures by NMR.

An all-atom **G–G** macrocycle model was built using Antechamber^17^ with atomic point charges parameterized using the restrained electrostatic potential (RESP) approach^18^ based on HF/(6-31)G* calculations performed using Gaussian 16.^19^ After an equilibration protocol (see details in the Computational Methods section), a 200 ns-long WT-MetaD simulation was run using D1 and D2 as collective variables (CVs) which discriminate between folded and open conformations. All the simulations were carried out using GROMACS^20^ patched with PLUMED.^21,22^ After reaching convergence, the WT-MetaD results were reweighted using the Tiwary–Parrinello free energy estimator^23^ to obtain free energy surfaces (FESs) (Figure 1). For the CVs, descriptors of the conformational changes in the macrocycle structure, we use the distances indicated as D1 and D2 of **G–G** in Chart 1 (in red). These two distance parameters, D1 and D2, were found to work well as CVs in the MetaD simulations. Distance D1 samples the folding/unfolding of **G–G** (recrossing between the folded and extended macrocycle conformations). Distance D2 probes the repulsion/interaction between the protonated triazines (significant for the folding/unfolding of **G–G**).

During the WT-MetaD simulation, the system is biased to explore all the possible combinations of D1 and D2, where the macrocycle visits different conformations. From well-converged WT-MetaD, we reconstruct complete FESs describing the conformations accessible by the macrocycle under various conditions. Figure 1 (left) shows the FESs, projected on the D1–D2 plane, obtained from the WT-MetaD simulations of the macrocycle in different solvents. All the conformations that belong to every identified minimum can be extracted using a clustering algorithm (based on the all-atom root mean square deviation of all the conformations), yielding a statistically significant centroid of the conformational ensemble. The FESs also provide information on the magnitude of the energetic barriers between the dominant minima. The data obtained by our calculations offer a valuable qualitative insight into the structural dynamics of this macrocycle and into its “chameleon-like” behavior.

WT-MetaD reveals a FES with relatively shallow minima. That is, in water, the regions indicated **A/A′** and **B** are only 6 kT (<4 kcal/mol) lower than the highest-energy conformations. Although the height of barriers estimated via WT-MetaD should be considered qualitatively, the obtained barrier heights are consistent with expectation (e.g., for what pertains to the interconversion of these isomers via rotation around σ-bonds of the aliphatic domain between the hydrazone and amino acid). Similar “shallow minima” are essentially present in all the FESs that are calculated herein, leading us to conclude that **G–G** exists as a highly dynamic molecule under the simulated conditions. At room temperature, we would expect to see ^1^H NMR reflecting resonances corresponding to the average of these conformations with rapid interconversion between **A/A′** and **B**. Indeed, we see only one set of resonances in all of these solvents.

Closer inspection of the FESs, however, suggests that the solvent may play some role in conformation preference. For example, the surfaces that describe the conformational landscape in water and methanol are quite similar. At low values of D1 and D2 (lower left corner of the FESs), a pair of enantiomeric, folded structures, **A** and **A′**, emerge that show extensive π–π overlap. At higher values of D1 and D2 (upper right in the FESs), an extended structure, **B**, is seen. Slight differences in the shape and centers of these minima are revealed by representative structures, which show that the regions of π–π overlap can easily shift and twist, generating a cluster of conformations around **A/A′** which are relatively similar in free energy.^24^ The shapes and centers of the minima for structures associated with **B** are more conserved, which reflects its open, extended conformation. In order to facilitate the comparison of the behavior of **G–G** in water with that in acetonitrile and dimethyl sulfoxide (DMSO) solvents, we shift from examination of the FESs to the probability distributions that can be calculated from them.

From the FESs, it is also possible to obtain information on the relative probabilities [*P*(*r*)] for the populations of all sampled conformers based on their relative free energy levels (Figure 1: right) as in eq 1
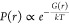
1

The probability maps included in Figure 1 (right) lead us to the conclusion that a folded conformation (**A/A′**) is populated and present in all solvents, meaning that **G–G** folding is possible in all cases. Methanol and acetonitrile show population densities that are similar in dimension/shape. DMSO shows two density peaks corresponding to **A/A′**. Water shows a larger density peak which suggests the additive sum of the previous three leading to the conclusion that in water, the folded state is more dynamic/diverse. In methanol, we observe that the peaks of the folded and unfolded conformers are very similar, which suggests that in this solvent, **G–G** is better solvated, and it has equal probability to exist in the folded or unfolded state. These observations must be tempered with recognition that barriers between these structures on the surface are low and the experimental observation that a single set of resonances is seen in the ^1^H NMR spectra of these species (a signature of rapidly interconverting conformers). Even in methanol, where a second, extended conformation (B) is populated, only one set of resonances is observed by NMR, which suggests that the folded and unfolded conformations interconvert.

Table 1 shows the estimated **A/A′** and **B** populations calculated in four solvents. The designation “other” refers to all other sampled conformations obtained by summing the remaining FES points. The areas associated with **A/A′** and **B** were calculated by including all conformations with Δ*G* < 3 kT from the minima.

**Table 1:** Populations (in %) of Conformers as Predicted by WT-MetaD Simulations in Various Explicit Solvents

isomer	water	DMSO	MeCN	MeOH
**A/A′**	80	52	65	43
**B**	5	11	2	20
other	15	37	33	37

The predictions from WT-MetaD simulations, both in terms of the structure and dynamic behavior of **G–G**, are supported by the experiments. A folded structure for a derivative of **G–G** is observed using single-crystal X-ray diffraction.^25^

Consistent with the prediction of a compact structure, the solid-state structure is found to be symmetric and shows extensive π–π stacking of the triazine ring upon the hydrazone. Both 1D and 2D NMR spectroscopy experiments further support the evidence from the WT-MetaD simulations. At room temperature, the NMR spectra of **G–G** in DMSO-*d*_6_, CD_3_OD/H_2_O, CD_3_CN, and D_2_O/H_2_O show a single set of resonances corresponding to equivalent environments for each half of the symmetric dimer.

**Figure 2:**
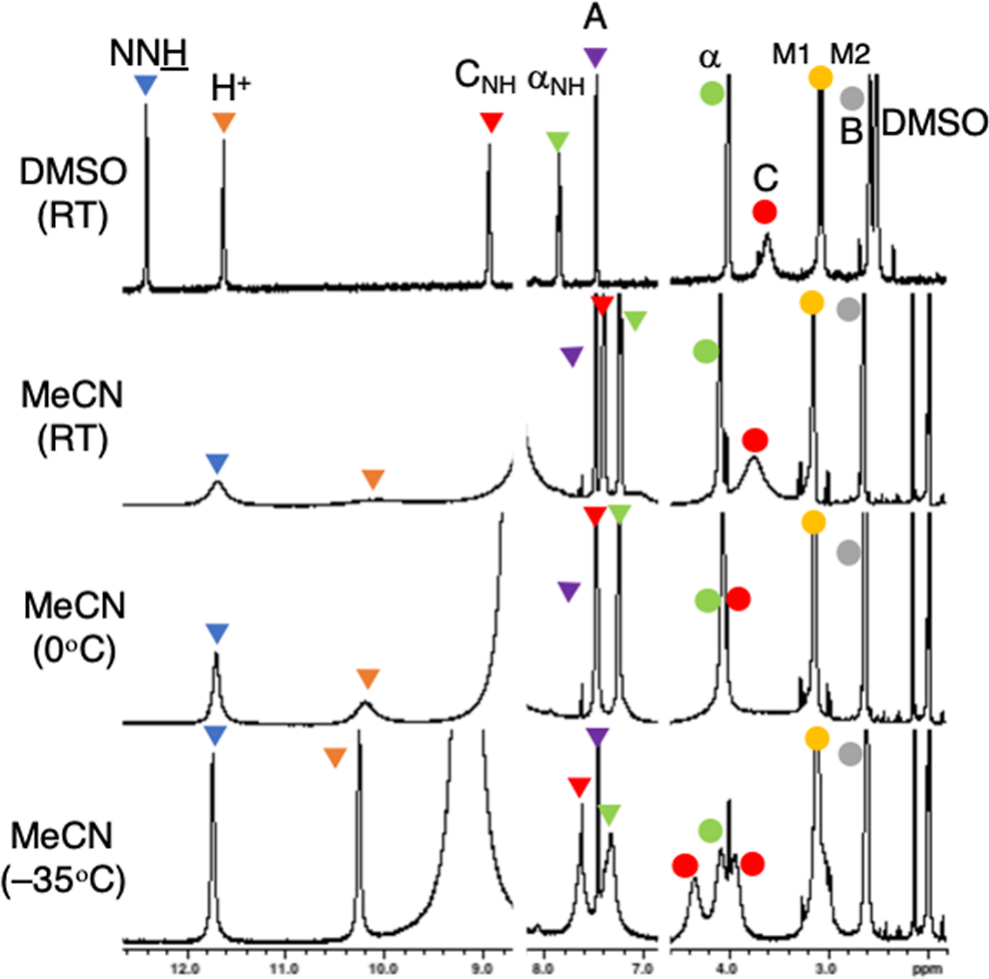
Variable-temperature spectra of **G–G** in MeCN-*d*_3_. At −35 °C, molecular motion slows, and the enantiotopic methylenes α and C become diastereotopic due to conformational chirality, while downfield resonances remain unaffected.

Consistent with prediction, the ^1^H NMR spectra show similar chemical shifts across the solvents surveyed. The spectrum in DMSO-*d*_6_ (Figure 2, top) is well resolved except for the broad resonance of C, a feature we attribute as indicative of the locus for dynamic motion for interconversion of **A** ⇔ **B** ⇔ **A′**. Critical rOes include H^+^ to α-NH (site of protonation), NHN=CH– (*trans*-hydrazone), and C–NH to both α and H_3_CNCH_3_ (amide orientation).

The most interesting experimental support for the dynamic interconversion of A ⇔ B ⇔ **A′** derives from low-temperature NMR obtained in CD_3_CN (Figure 2). As the temperature is decreased, in the fingerprint region of the spectra from 7 to 13 ppm, the number of resonances remains constant. Rapidly exchanging, downfield NH protons sharpen, and other resonances—that is, the hydrazone methine proton (NHN=CH−) and amide NH—broaden as expected due to viscosity. Upfield, however, the α and C methylene protons of **G–G** decoalesce.

This markedly different behavior is not consistent with the emergence of a new conformation per se because we would expect the chemical environment of all resonances to be affected. Instead, this behavior reflects the emergence of diastereomers resulting from the slowing of the interconversion process A ⇔ B ⇔ **A′**. That is, methylenes α, B, and C are enantiotopic. They are interconverted by a transition between enantiomeric conformations of the macrocycle at higher temperatures. However, at −35 °C, the folded conformations persist longer than the NMR timescale, and we observe the emergence of conformational chirality—reviewed recently by De Riccardis.^26^ Accordingly, these enantiotopic methylenes are rendered diastereotopic and are resolved in the spectra. At −20 °C in CD_3_OD, the **B** methylene becomes diastereotopic as well.

## Conclusions

In this work, we show how WT-MetaD simulations can provide precious qualitative insights, useful to predict and characterize the key structural and dynamical features of the **G–G** macrocycle in various solvents, providing evidence of the “chameleon-like” dynamic behavior of such a macrocycle under experimentally relevant conditions. As increased attention turns to controlling macrocycle conformation,^27,28^ identifying accessible conformations and characterizing the barriers of interconversion between them offer compelling motivation for its broadening application.^29,30^ Its use to survey the effects of substitution on different sites on this class of macrocycles is ongoing.

## Computational Methods

### Parametrization, Minimization, and Equilibration

The atomistic **G–G** macrocycle model was parametrized using Antechamber^17^ according to the Generalized Amber Force Field (GAFF)^31^ with atomic point charges parameterized using the RESP approach^18^ based on HF/(6-31)G* calculations performed using Gaussian 16.^19^ The **G–G** macrocycle model was inserted in a periodic simulation box that was filled with different types of explicit solvents. In detail, we obtained atomistic models for the **G–G** macrocycle solvated in explicit water (TIP3P),^32^ DMSO,^33^ acetonitrile,^34^ or methanol.^35^ All simulations were run under periodic boundary conditions. The systems were initially minimized using the steepest descent algorithm for 2000 steps and then with a conjugate gradient algorithm for further 2000 steps. After minimization, each model system underwent preliminary MD simulations to allow the systems to reach a temperature of 298 K (under *NVT* conditions for 5 ns) and a pressure of 1 bar (under *NPT* conditions). In the *NPT* MD phase, we used the Berendsen barostat^36^ for 1 ns, imposing a pressure of 1 bar (coupling time of 1 ps), followed by the last *NPT* equilibration run using the Parrinello–Rahman barostat^37^ set at 1 bar (coupling time of 1 ps) for 1 ns. All simulations ran in this work used a velocity-rescale thermostat^38^ (at 298 K, a coupling time of 0.2 ps) and a simulation time step of 2 fs, allowed by the use of LINCS^39^ constraints for all the bonds that involve hydrogen atoms. All simulations were performed with GROMACS 2021.2^20^ patched with PLUMED 2.7.^21,22^

### Well-Tempered Metadynamics Simulations

For all the four solvents considered herein, we performed 200 ns-long^16^ WT-MetaD simulations using the same MD parameters as those used in the last equilibration step (see the previous section). During this simulation time, all systems reached convergence. As descriptors of the **G–G** macrocycle conformations (and conformational changes), we used two CVs, namely, distances D1 and D2 in Chart 1 in main text. For both variables, we set a sigma of 0.025 nm, a Gaussian height of 1.2 kJ/mol, and a bias factor of 25. During the WT-MetaD runs, we deposited a Gaussian on the landscape every 1 ps. After the WT-MetaD simulations, a reweighting procedure was performed using the Tiwary–Parrinello estimator.^23^ For the analyses of the FESs (and population probabilities), we considered the last 100 ns of our simulations.

## Supporting Information Available

Detailed experimental procedures, spectra, and additional computational calculations (PDF)

## Notes

Complete input, simulation, and data analysis files are available at the Zenodo repository https://doi.org/10.5281/zenodo.6967170.
