# Supplementary material for: Well-Tempered Metadynamics Simulations Predict the Structural and Dynamic Properties of a Chiral 24-Atom Macrocycle in Solution
Source: ACS Omega. 2022 Aug 8;7(34):30291–6. doi: 10.1021/acsomega.2c03536 (PMC9434777; doi:10.1021/acsomega.2c03536)
Supplement: Supplementary file 1 — ao2c03536_si_001.pdf [file ao2c03536_si_001.pdf]

SUPPORTING INFORMATION

Well-tempered Metadynamics Simulations Predict the Structural and Dynamic Properties of a Chiral 24-Atom Macrocycle in Solution

Riccardo Capelli,[1] Alexander J. Menke,[2] Hongjun Pan,[3] Benjamin G. Janesko,[2] Eric E. Simanek[2,*] and Giovanni M. Pavan[1,4,*]

[1]Department of Applied Science and Technology, Politecnico di Torino, 10129 Torino, Italy
Email: giovanni.pavan@polito.it
[2]Department of Chemistry & Biochemistry, Texas Christian University, Fort Worth TX 76129
Email: e.simanek@tcu.edu
[3]Department of Chemistry, University of North Texas, Denton TX 76129
[4]Department of Innovative Technologies, University of Applied Sciences and Arts of Southern Switzerland, Polo Universitario Lugano, 6962 Lugano-Viganello, Switzerland

TABLE OF CONTENTS

I. Computational SI

II. Chart S1. Compounds described in the supporting material

III. General Experimental Details

IV. Experimental Details for Synthesis of Relevant Compounds

V. Spectra

Figure S1. ^{1}H NMR spectrum of **G-G** in DMSO-d_6
Figure S2. ^{1}H NMR spectrum of **G-G** in D_2O
Figure S3. Variable Temperature ^{1}H NMR spectrum of **G-G** in MeOD-d_4
Figure S4. Variable Temperature ^{1}H NMR spectrum of **G-G** in MeCN-d_3
Figure S5. ^{13}C NMR spectrum of **G-G** in DMSO-d_6
Figure S6. COSY NMR spectrum of **G-G** in DMSO-d_6
Figure S7. COSY NMR spectrum of **G-G** in MeCN-d_3 at 65 ^{o}C
Figure S8. The rOesy NMR spectrum of **G-G** in DMSO-d_6
Figure S9. HSQC NMR spectrum of **G-G** in DMSO-d_6
Figure S10. ^{1}H NMR spectrum of **3** in DMSO-d_6
Figure S11. ^{13}C NMR spectrum of **3** in DMSO-d_6
Figure S12. ^{1}H NMR spectrum of **2** in DMSO-d_6
Figure S13. ^{13}C NMR spectrum of **2** in DMSO-d_6

SECTION I – Computational Methods

Classical and enhanced sampling simulations

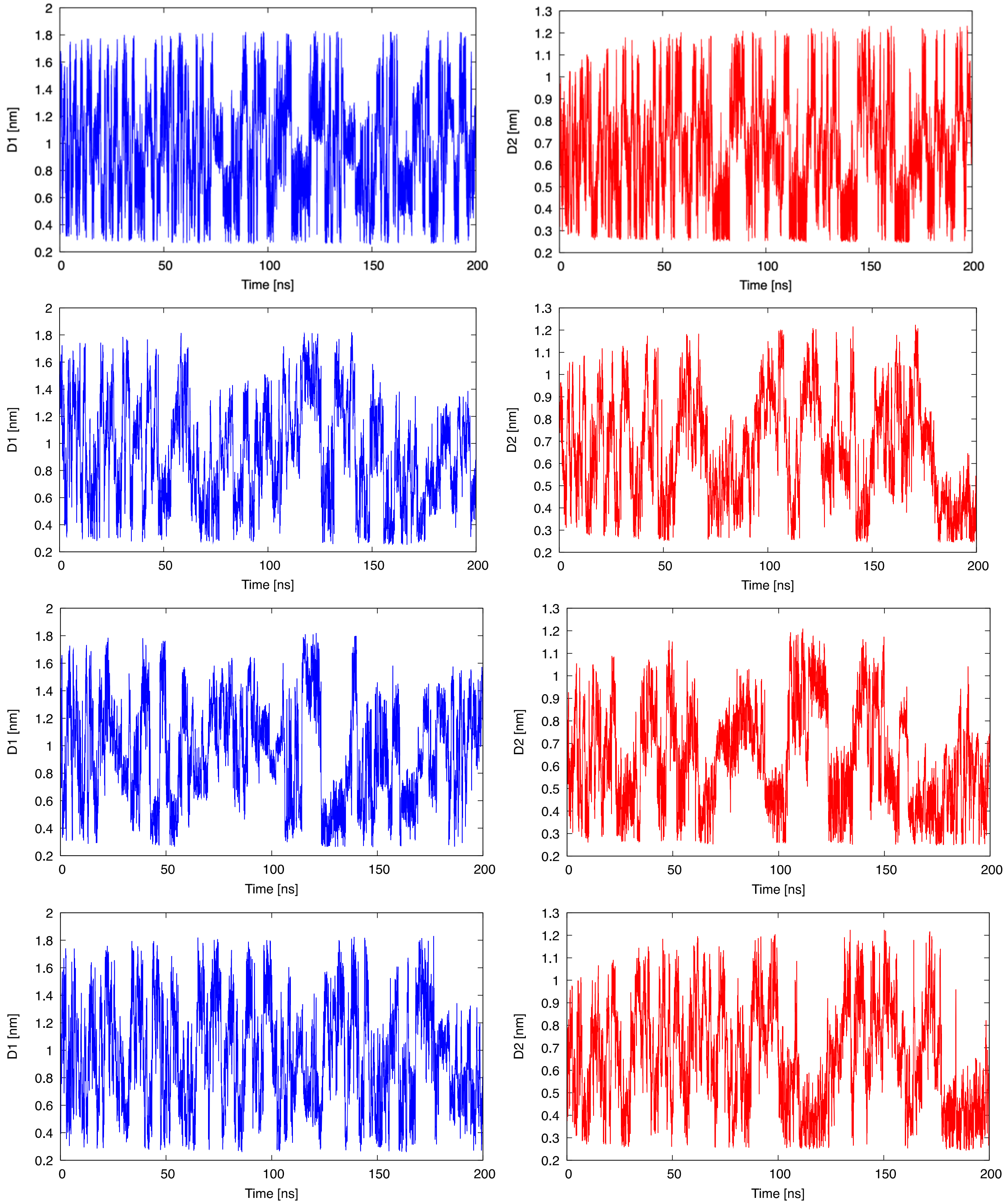

Figure S1: Time evolution of D1 and D2 (CVs) during the WT-MetaD simulation of G-G macrocycle in water (1st row), DMSO (2nd row), MeCN (3rd row), and MeOH (4th row). The diffusive behavior of the system in the CVs space corroborates the hypothesis of convergence.

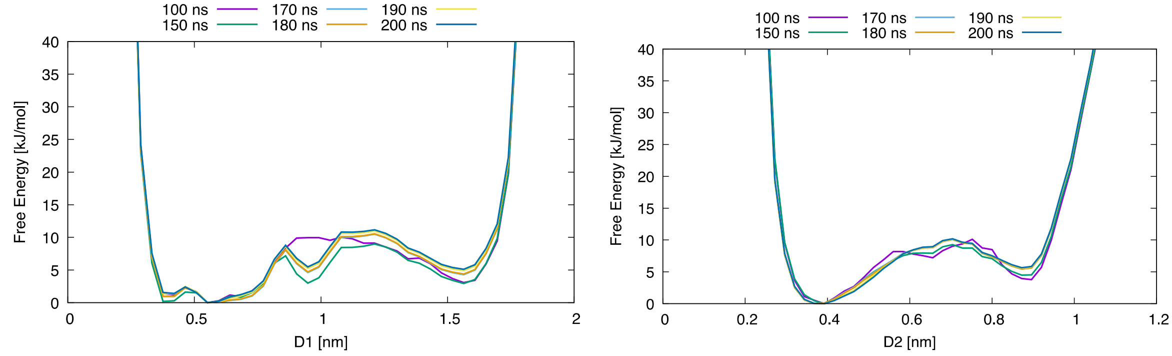

Figure S2: Time evolution of the G-G macrocycle in water FES during the WT-MetaD simulations in the last 30 ns projected along the single CVs D1 and D2. We can observe a substantial convergence in both the projections.

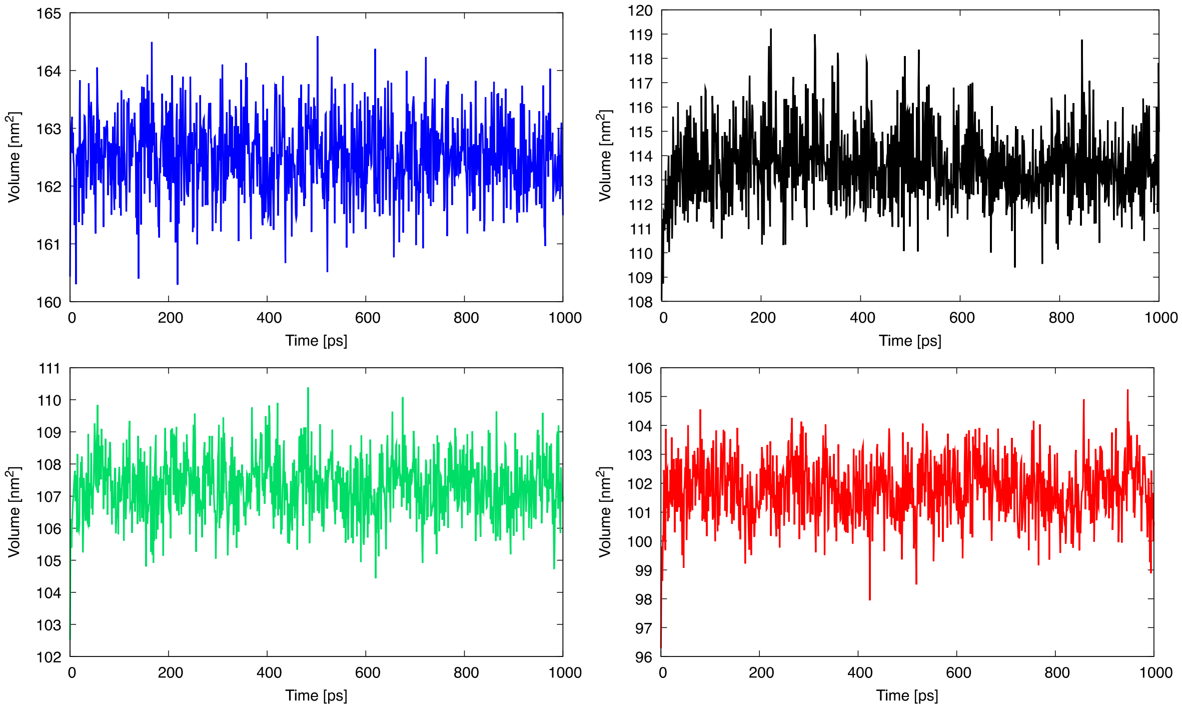

Figure S3: Convergence plot for NpT equilibration for all the 4 systems in analysis: water (top left), DMSO (top right), MeCN (bottom left), and MeOH (bottom right).

SECTION II

Chart S1. Compounds described in the supporting material

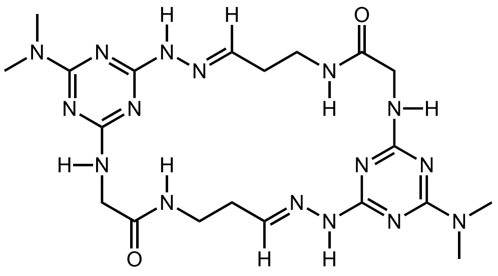

G-G

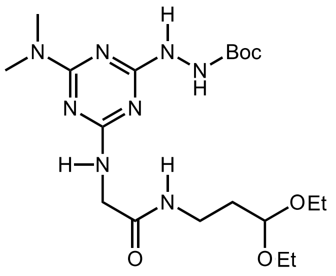

2

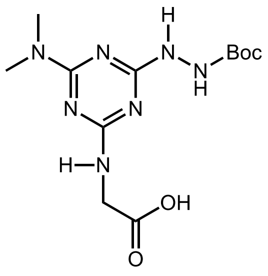

3

SECTION III – General Experimental Details

NMR Spectroscopy. Room temperature ^{1}H NMR spectra were recorded on a 400 MHz Bruker Avance spectrometer. Chemical shifts for ^{1}H NMR spectra (in parts per million) referenced to a corresponding solvent resonance (e.g. DMSO-d_6, $\delta = 2.52$ ppm). $^{13}C\{^{1}H\}$ NMR spectra were recorded on the same 400 MHz Bruker spectrometer referenced to corresponding solvent resonance. All 2D spectra were taken on the 400 MHz Bruker Avance relative to corresponding solvent resonances. Low temperature spectra were acquired on a 500 MHz Varian NMR spectrometer at the University of North Texas in Denton. Identification of NMR signals are as follows: s = singlet, d = doublet, t = triplet, and m = multiplet. NMR solvents were deuterated and purchased as a bottle or ampule.

General Chemistry. Flash chromatography experiments were carried out on silica gel with a porosity of 60Å, particle size 50–63 μm, surface area 500 – 600 m^2/g, a bulk density of 0.4 g/mL and a pH range of 6.5 – 7.5. Dichloromethane/methanol was used as the eluent for chromatographic purification. Thin-layer chromatography experiments were carried out in sealed chambers and visualized with UV or submersion in ninhydrin (1.5g ninhydrin in 100mL of *n*-butanol and 3.0mL acetic acid) followed by heating. Excess solvents were removed via rotary evaporation on a Buchi Rotavapor RII with a Welch Self-Cleaning Dry Vacuum System. All workup and purification procedures were carried out with reagent-grade solvents under ambient atmosphere.

SECTION IV – Experimental Details

*Synthesis of the glycine acid intermediate, **3**.* Intermediate **3** was prepared in one-pot by sequential addition to cyanuric chloride as illustrated and described below.

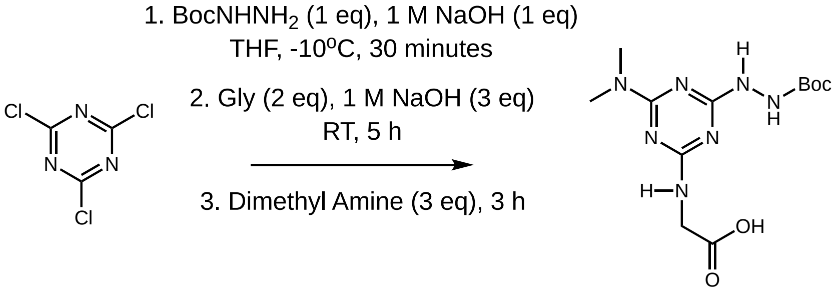

Cyanuric chloride (1.0 g, 5.4 mmol) was added rapidly as a solid to a stirring flask containing 25 mL of THF that was previously cooled to –10 °C using a dry ice and acetone bath. The temperature was maintained at –10 °C. Upon dissolution (which was immediate), a 25 mL solution of BOC-hydrazine (0.72 g, 5.4 mmol) in THF (0.2 M) was added dropwise over 2 minutes. Over the course of the addition, the solution turned a very pale yellow. After the addition was complete 5.4 mL of 1 M NaOH (5.4 mmol) was added over 1 minute via pipette. After 30 minutes, thin layer chromatography (1:9 MeOH:EtOAc) confirmed that a single product (R_f = 0.7) was observed using either short wave UV irradiation or using ninhydrin (yellow spot). At this time, the ice bath was removed, and the solution allowed to slowly warm to room temperature.

A solution of glycine (0.81 g, 10.8 mmol) in 10 mL H_2O and 10.8 mL of 1 M NaOH (required to dissolve the glycine) was added dropwise over 2 min while at room temperature. The reaction mixture was pH of 8 after the addition. After 1 hour, the solution measured pH 6 and another equivalent of 1 M NaOH was added to return to a pH of 8. The solution started a pale yellow and turned bronze in color. After 5 h, thin layer chromatography (1:9 MeOH:EtOAc) showed the starting material (R_f = 0.7) disappeared and a new spot at R_f = 0.05 appeared using both short wave UV irradiation or ninhydrin (yellow spot).

A 40% aqueous solution of dimethylamine (1.84 g, 16.3 mmol) was added dropwise over three minutes. Immediately following addition, the solution was measured to be pH 9. The reaction was stirred for another 3 h at room temperature. Thin layer chromatography (1:30:70 acetic acid:methanol:chloroform) showed a new spot at R_f = 0.5. The reaction was acidified to pH 4 and the solvent was removed by rotary evaporation. Dichloromethane and EtOAc was added to dissolve the organic-soluble product. Column chromatography (1:30:70 acetic acid:methanol:chloroform) showed yielded 25% pure material.

^{1}H NMR (DMSO-D_6, 400 MHz): 8.56 – 8.50 (m, 1H), 8.34 – 8.07 (m, 1H), 6.82 – 6.67 (m, 1H), 3.68 – 3.78 (m, 2H), 3.00 (s, 5H), 1.40 – 1.30 (m, 9H).
^{13}C{^{1}H} NMR (DMSO-D_6, 100 MHz): δ 173.1, 167.9, 166.0, 156.5, 43.4, 35.9, 28.6.

Synthesis of the monomer acetal, ***2.*** Intermediate **2** was prepared as illustrated and described below.

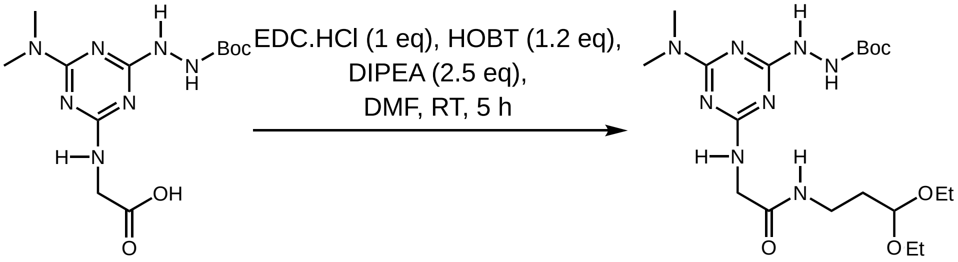

Glycine acid (0.05 g, 0.15 mmol), diethoxypropyl amine (0.022 g, 0.15 mmol), and HOBT (0.025 g, 0.18 mmol) were added sequentially to a stirring solution of 1.5 mL DMF at room temperature. Subsequently, DIPEA (0.049 g, 0.38 mmol) and EDC.HCl (0.035 g, 0.18 mmol) were added separately, neat. After 3 hours, thin layer chromatography (1:9 MeOH:CH_2Cl_2)

confirmed the single spot starting material (R_f = 0.25) had evolved into new spots by short wave UV irradiation. A single spot (R_f = 0.7 in 1:19 MeOH:CH_2Cl_2) stained yellow with ninhydrin. ninhydrin. The solvent was removed by rotary evaporation and silica gel chromatography was performed using a solven gradient of 2.5% to 5% MeOH in CH_2Cl_2. This effort yielded 0.02 g (29%) of pure material.

^{1}H NMR (DMSO-D_6, 400 MHz): δ8.55 – 8.51 (m, 1H), 8.37 – 8.08 (m, 1H), 7.71 – 7.61 (m, 1H), 6.89 – 6.80 (m, 1H), 4.46 (m, 1H), 3.84 – 3.72 (m, 2H), 3.52 (m, 2H), 3.41 (m, 2H), 3.09 (q, J = 2.4 Hz, 2H), 3.00 (s, 6H), 1.64 (m, 2H), 1.40 – 1.24 (m, 9H), 1.09 (t, J = 2.4 Hz, 6H).
^{13}C{^{1}H} NMR (DMSO-D_6, 100 MHz): δ 170.0, 167.6, 165.8, 155.7, 101.0, 79.0, 61.2, 44.5, 35.8, 35.1, 33.8, 28.6, 15.8.

<u>*Macrocyclization to yield **G-G***</u>. The macrocycle was obtained by the procedure illustrated and described below.

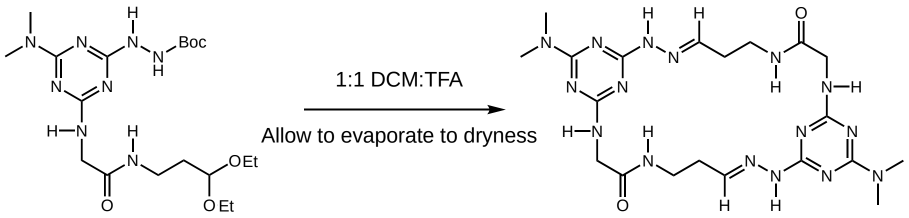

Acetal **2** (20mg) was dissolved in 1 mL of CH_2Cl_2 in a 3 mL vial equipped with a mini stir bar. Trifluoroacetic acid (1 mL) was added over 1 minute via pipette. The vial cap was perched on top of the vial without tightening to allow for slow evaporation. Evaporation occurred over the course of 5 days. The dried residue was then analyzed by NMR. The reaction can be described as quantitative.

^{1}H NMR (DMSO-D_6, 400 MHz): δ12.41 (s, 1H), 11.63 (s, 1H), 8.96 – 8.92 (t, J = 8 Hz, 1H), 7.86 – 7.83 (t, J = 8 Hz, 1H), 7.47 (s, 1H), 4.01 – 4.00 (d, J = 6 Hz, 2H), 3.60 (m, 2H), 3.09 (s, 3H), 3.05 (s, 3H), 2.57 (m, 2H).
^{13}C{^{1}H} NMR (DMSO-D_6, 100 MHz): δ 171.6, 161.8, 154.2, 153.7, 148.1, 44.2, 36.9, 36.8, 33.7, 32.1.
^{1}H NMR (D_2O, 400 MHz): δ 6.58 (s, 1H), 3.21 – 3.07 (m, 2H), 2.82 (m, 2H), 2.29 (s, 3H), 2.25 (s, 3H), 1.81 (s, 2H).
^{1}H NMR (CD_3CN, 400 MHz): δ 11.68 (s, 1H), 7.48 – 7.47 (t, J = 2.4 Hz, 1H), 7.41 (m, 1H), 7.24 – 7.21 (t, J = 6 H, 1H), 4.08 (d, J = 6 Hz, 2H), 3.73 (broad s, 2H), 3.14 (s, 3H), 3.12 (s, 3H), 2.64 – 2.61 (m, 2H).
^{1}H NMR (MeOD, 400 MHz): δ 8.80 (m, 1H), 7.85 (m, 1H), 7.42 – 7.41 (t, J = 2.4 Hz, 1H), 4.10 (s, 2H), 3.77 (m, 2H), 3.19 (s, 3H), 3.13 (s, 3H), 2.67 – 2.64 (m, 2H).

SECTION V – Spectra

The spectra appear on the following pages.

Figure S3. ^{1}**H NMR spectrum of G-G in DMSO-*d*$_6$.**

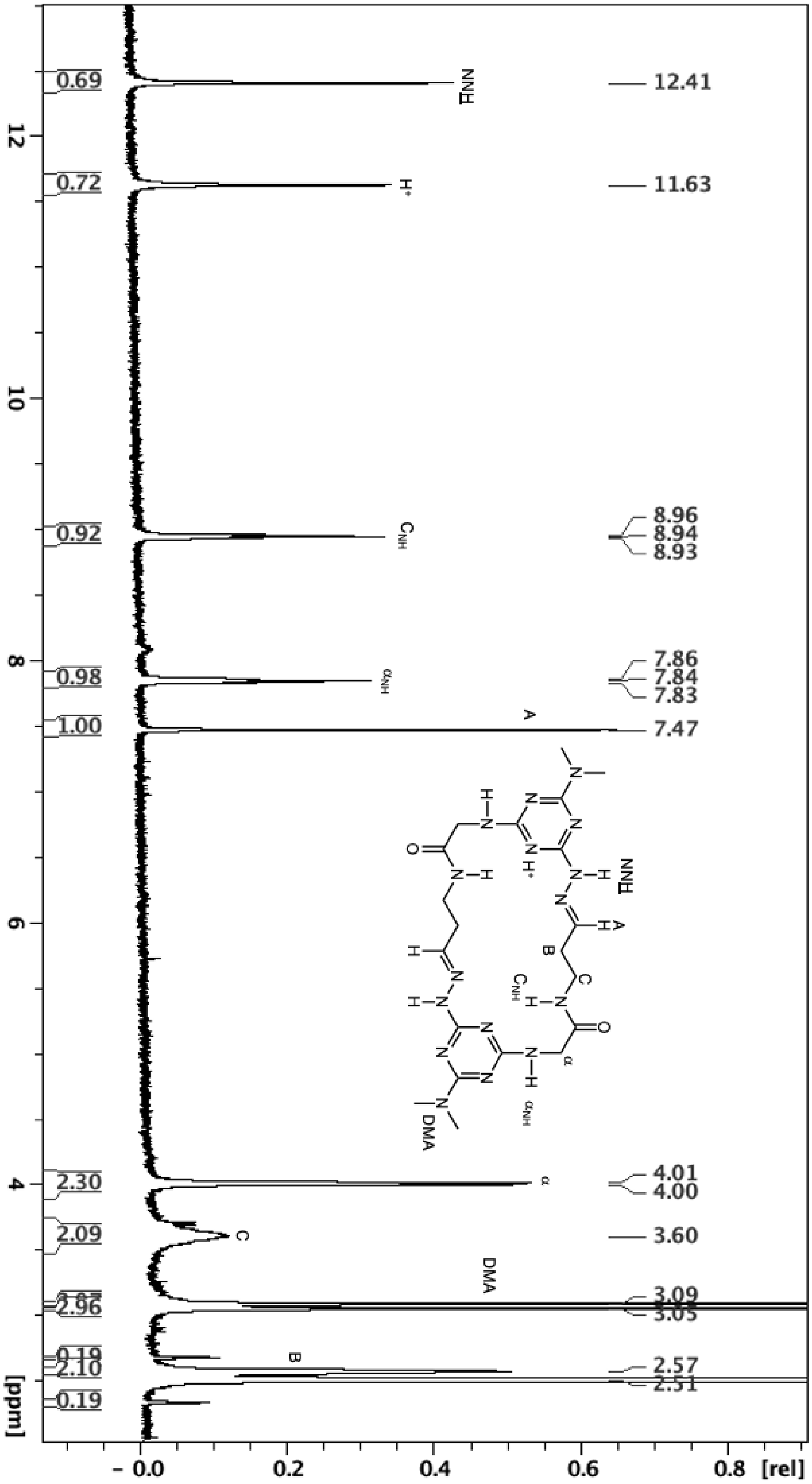

Figure S4. ^1H NMR spectrum of G-G in D_2O.

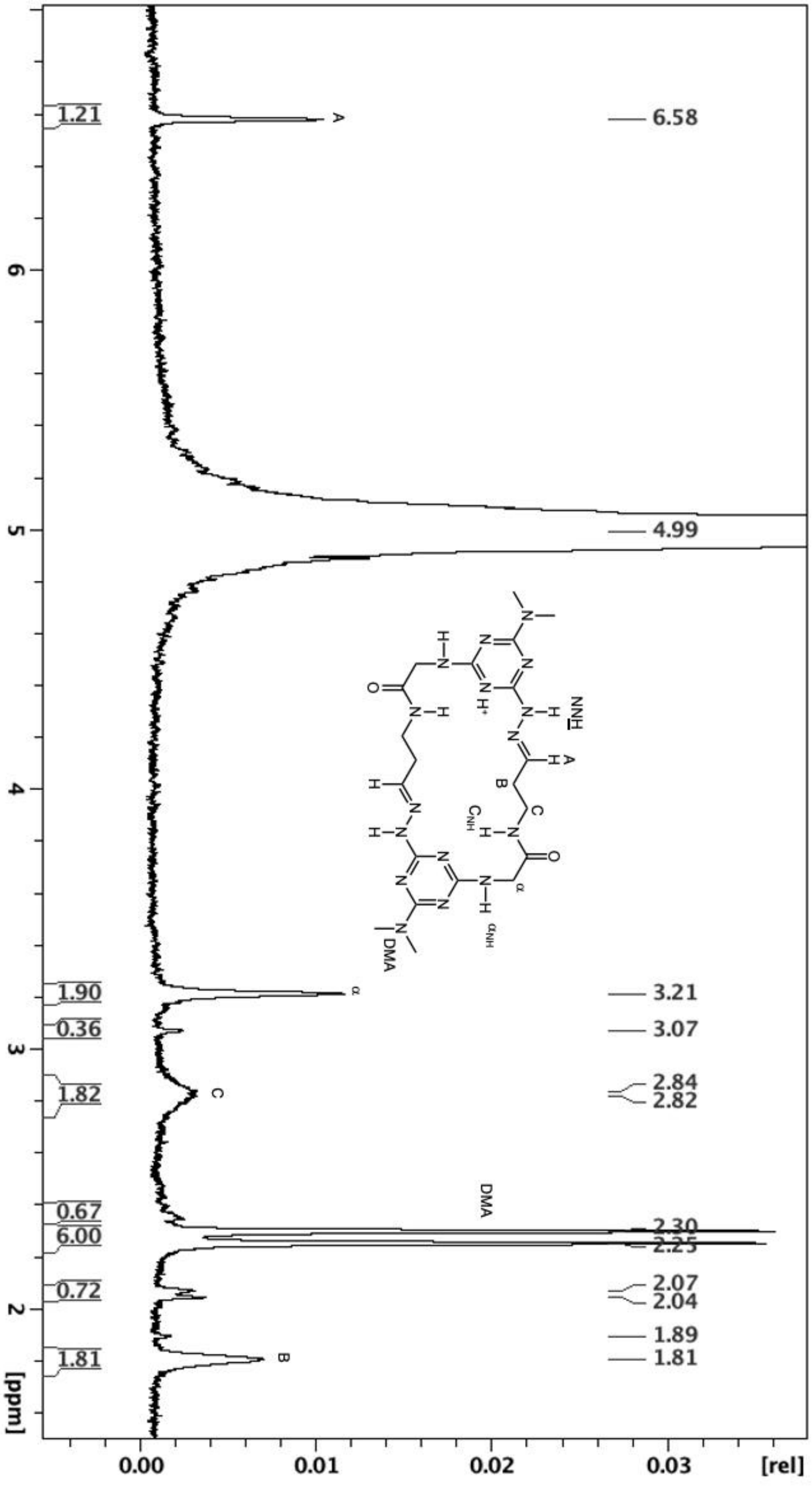

Figure S5. Variable Temperature ^{1}H NMR spectra of G-G in MeOD.

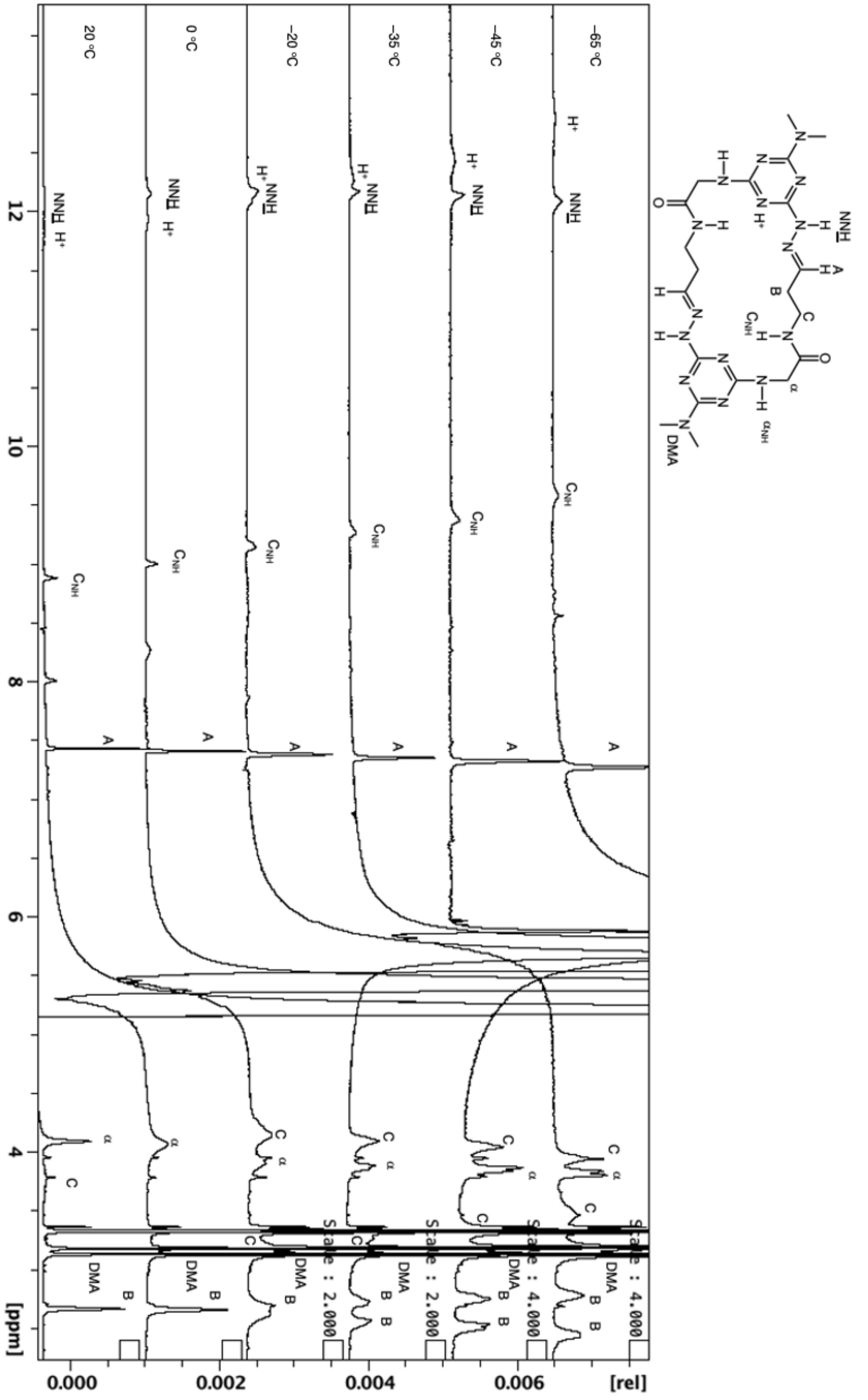

Figure S6. Variable Temperature ^{1}H NMR spectrum of G-G in MeCN-d_3.

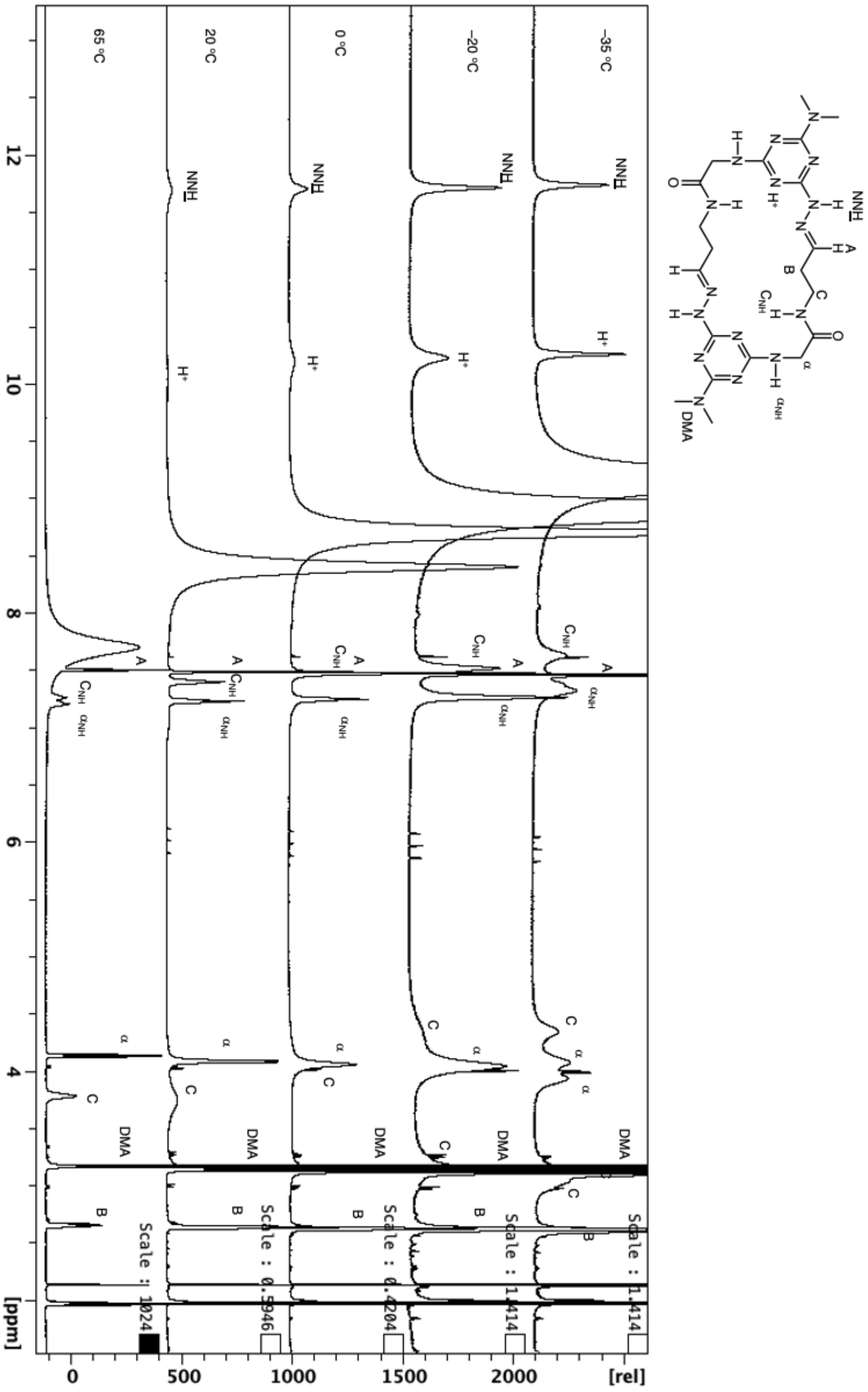

Figure S7. **^{13}C NMR spectrum of G-G in DMSO-d_6.**

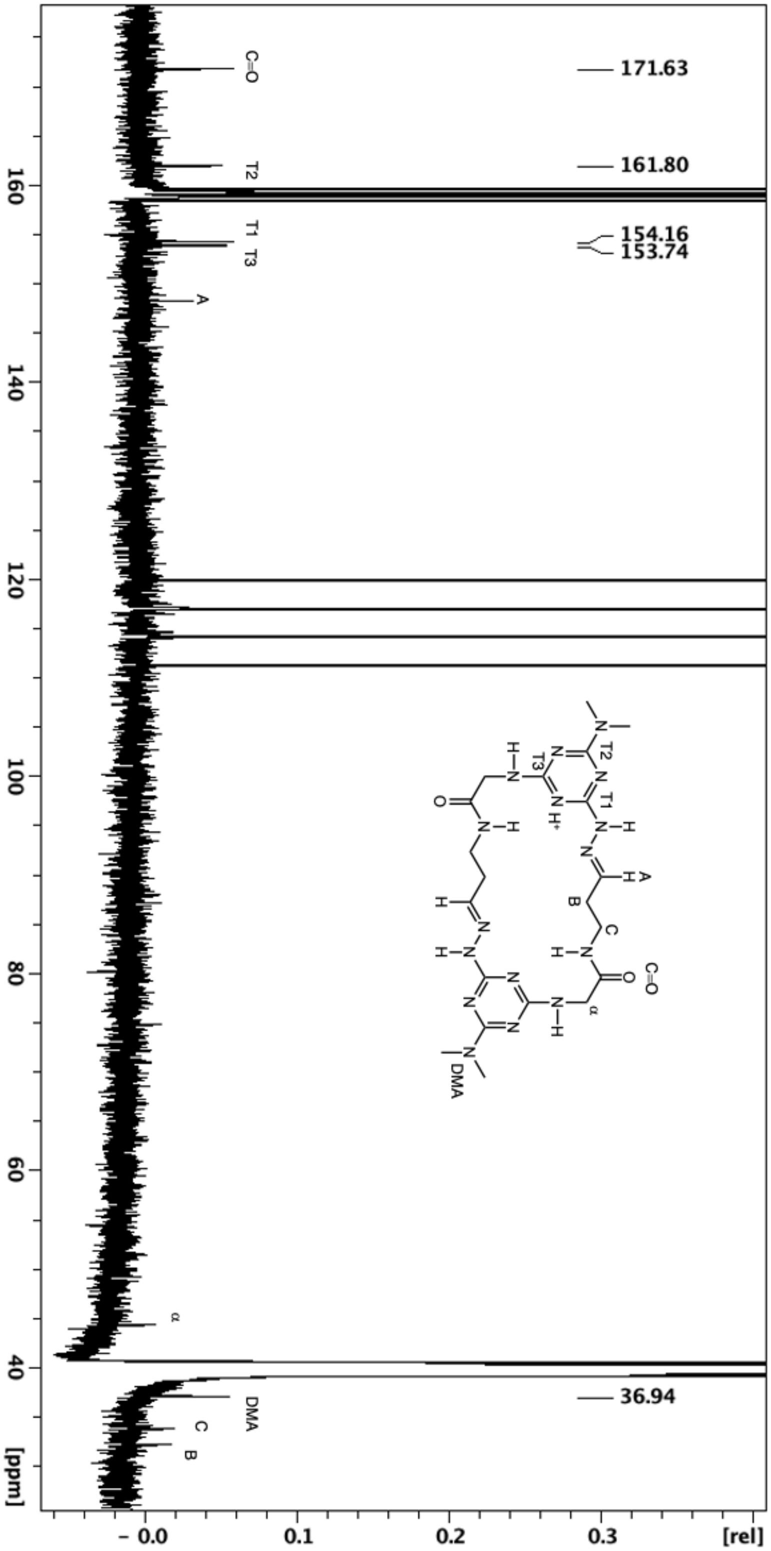

Figure S8. COSY NMR spectrum of G-G in DMSO-d_6.

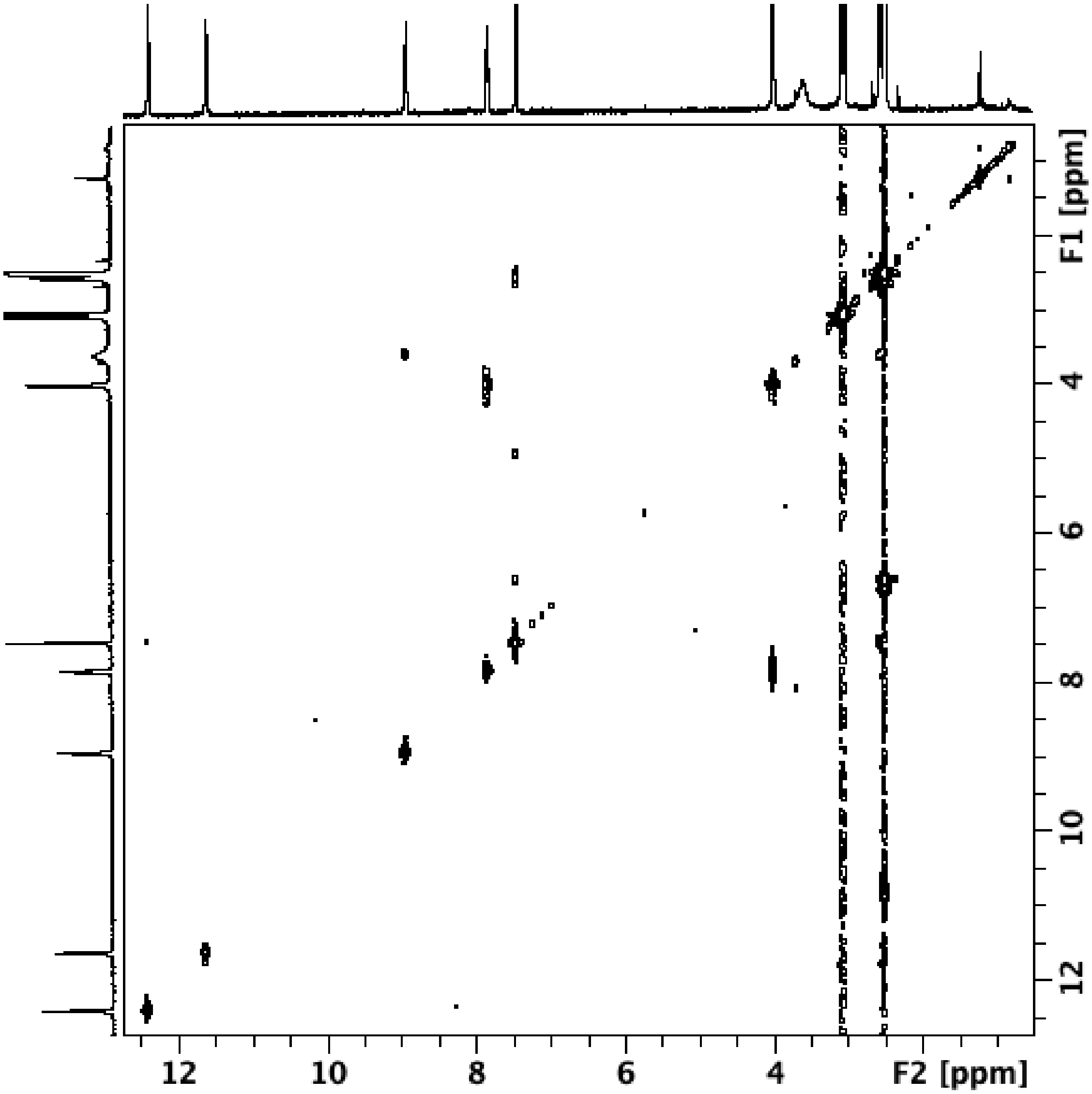

Figure S9. COSY NMR spectrum of G-G in MeCN-d_3 at 65 °C.

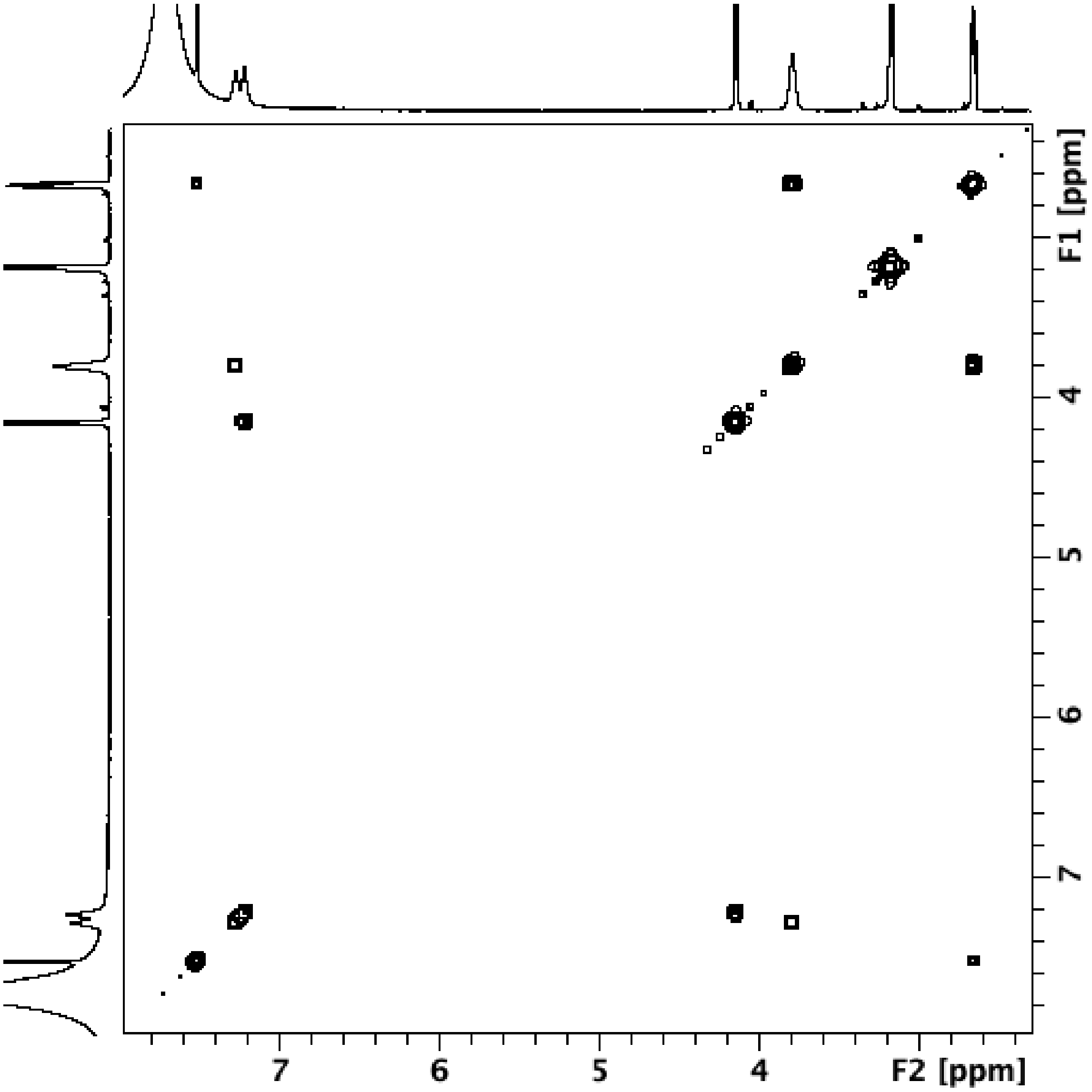

Figure S10. The rOesy NMR spectrum of G-G in DMSO-d_6.

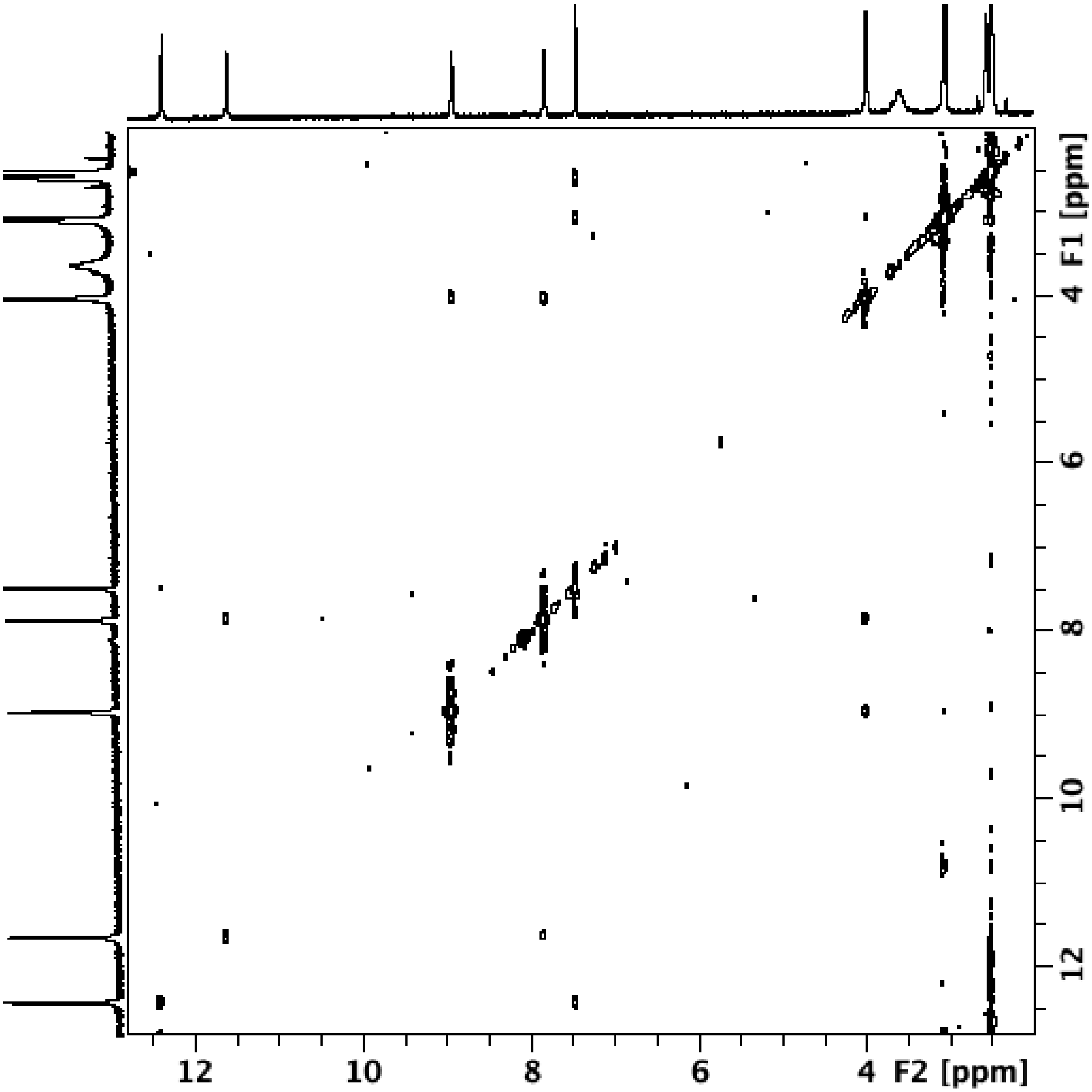

Figure S11. HSQC NMR spectrum of G-G in DMSO-d_6.

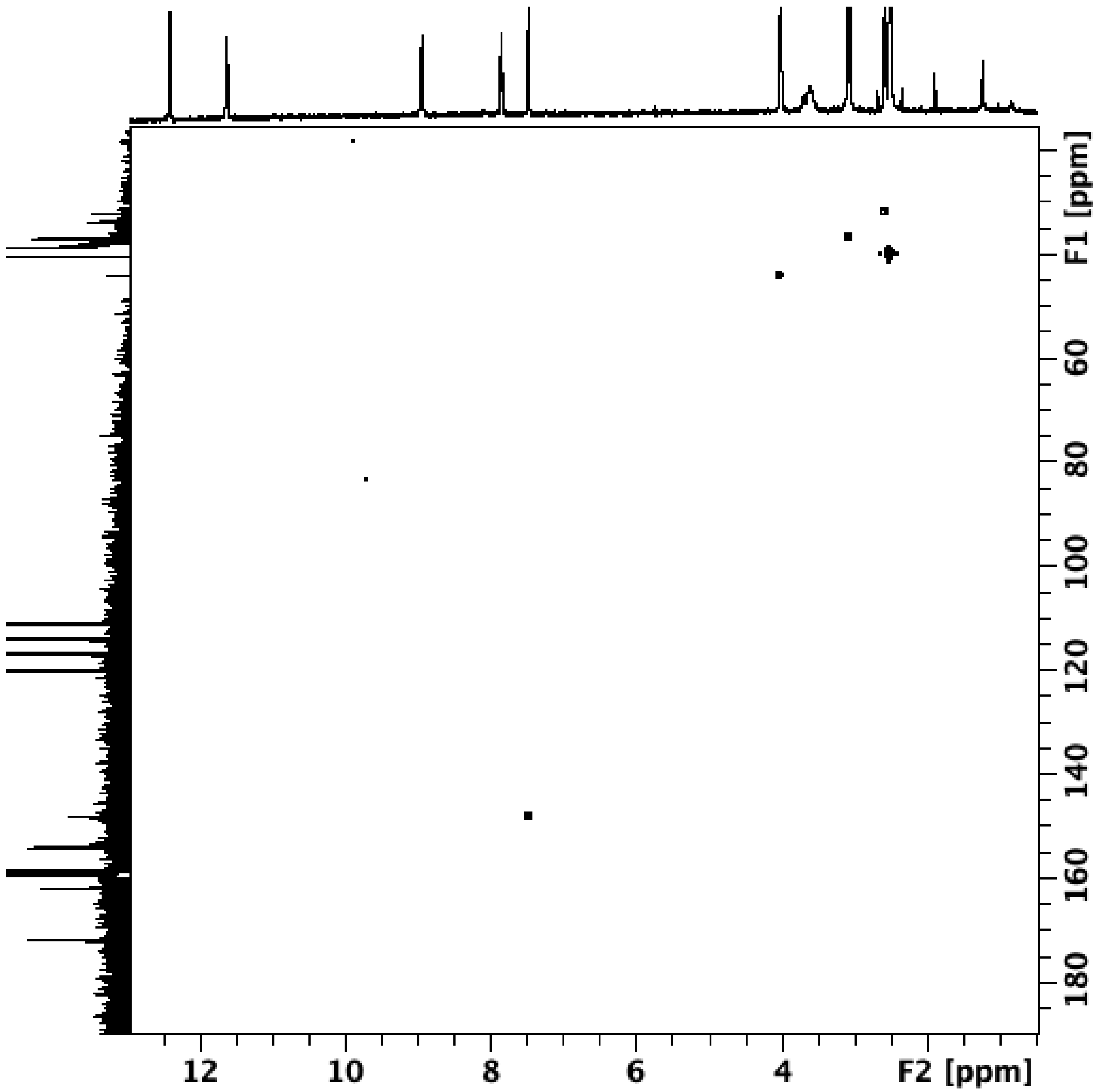

Figure S12. ¹H NMR spectrum of 2 in DMSO-d_6. Note the existence of multiple resonances for most signals due to the presence of rotational isomers resulting from the hindered rotation about the triazine-N bond.

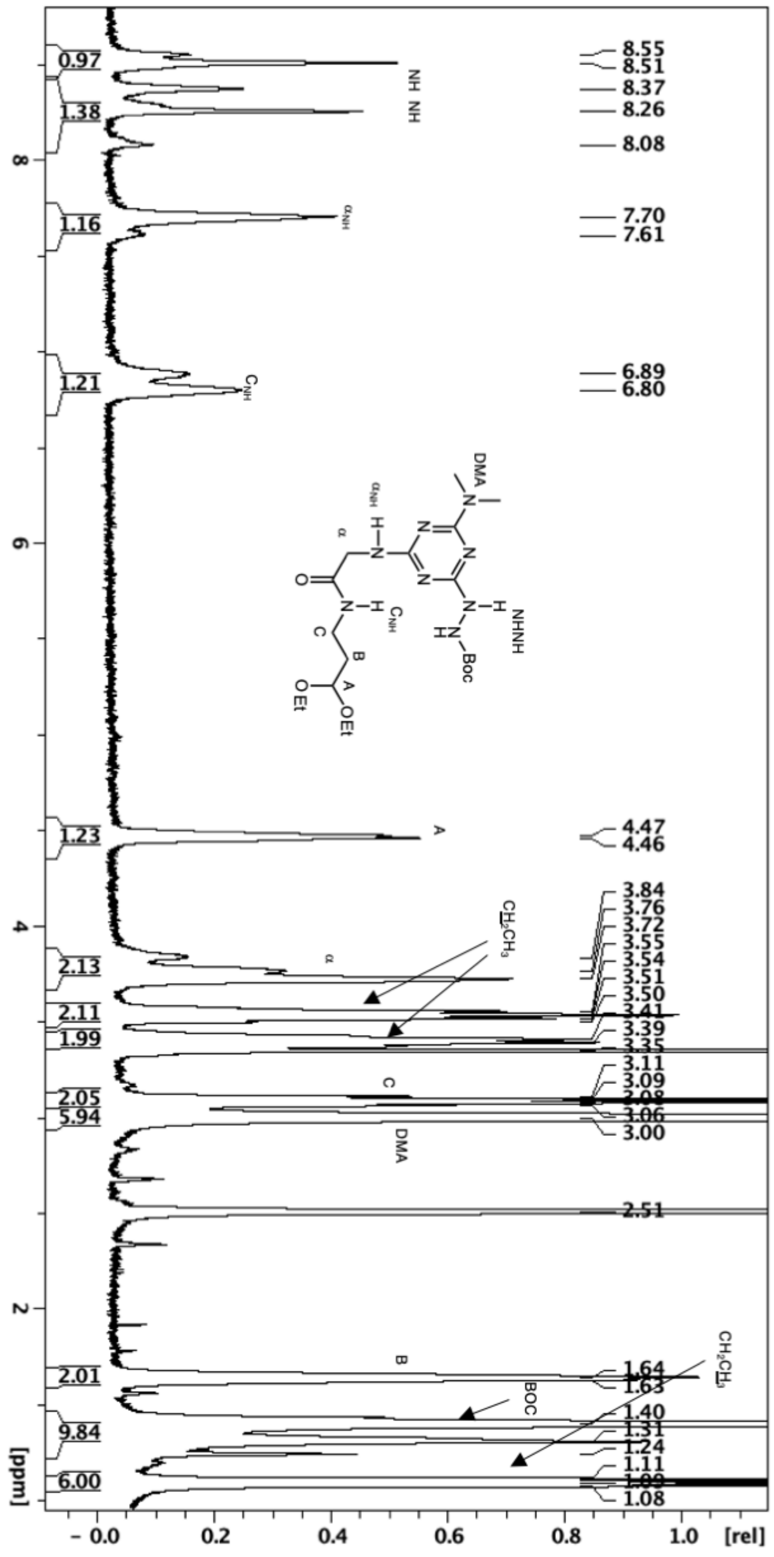

Figure S13. ^{13}C NMR spectrum of 2 in DMSO-d_6. The existence of rotational isomers resulting from the hindered rotation about the triazine-N bond preclude facile identification of triazine carbons which appear as broad 'lumps' and leads to multiple resonances for others.

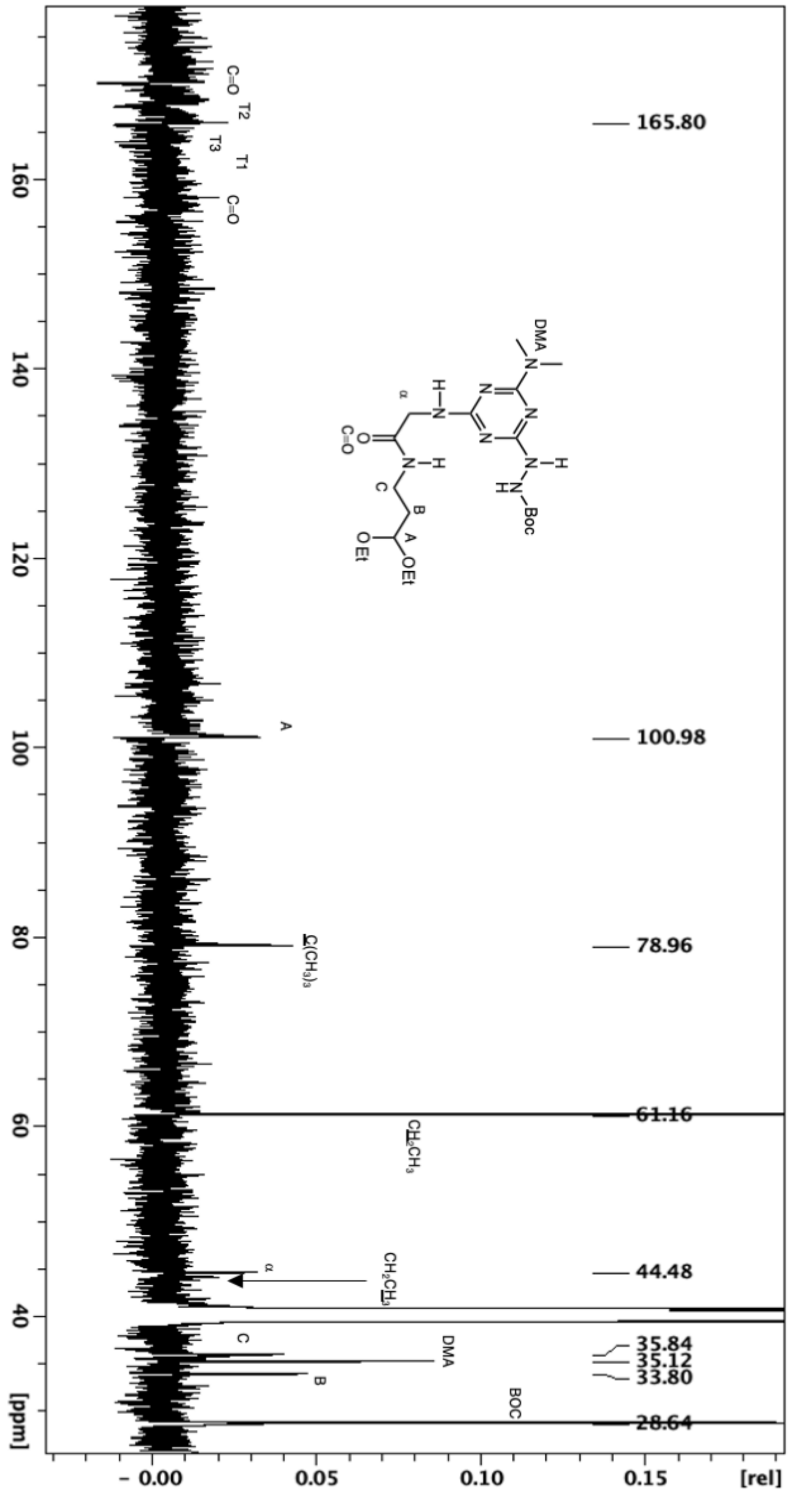

Figure S14. ^{1}H NMR spectrum of 3 in in DMSO-d_6. Note the existence of multiple resonances for most signals due to the presence of rotational isomers resulting from the hindered rotation about the triazine-N bond.

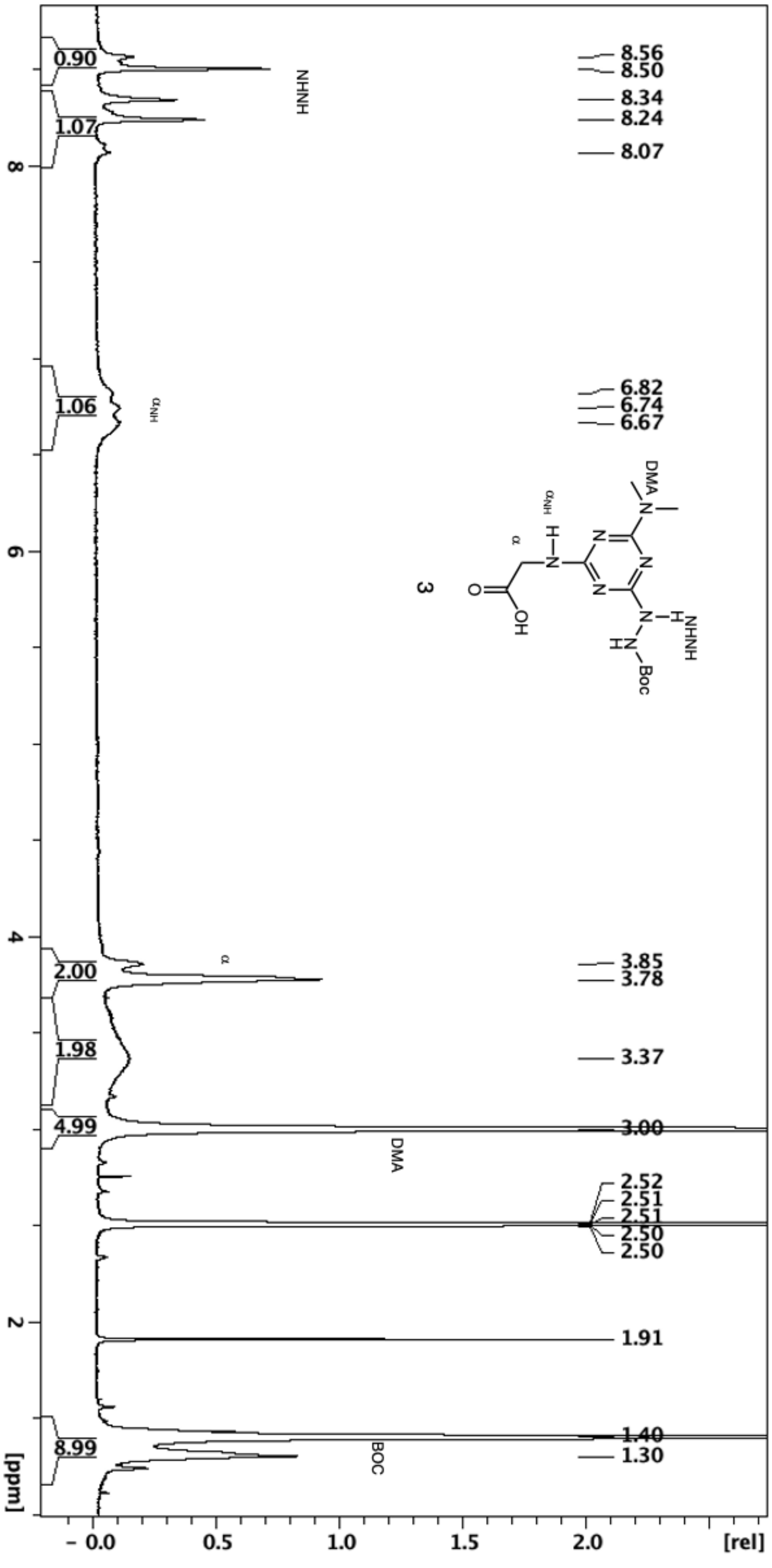

Figure S15. ^{13}C NMR spectrum of 3 in DMSO-*d_6*. The existence of rotational isomers resulting from the hindered rotation about the triazine-N bond preclude facile identification of triazine carbons which appear as broad and leads to multiple resonances for these carbons and others.

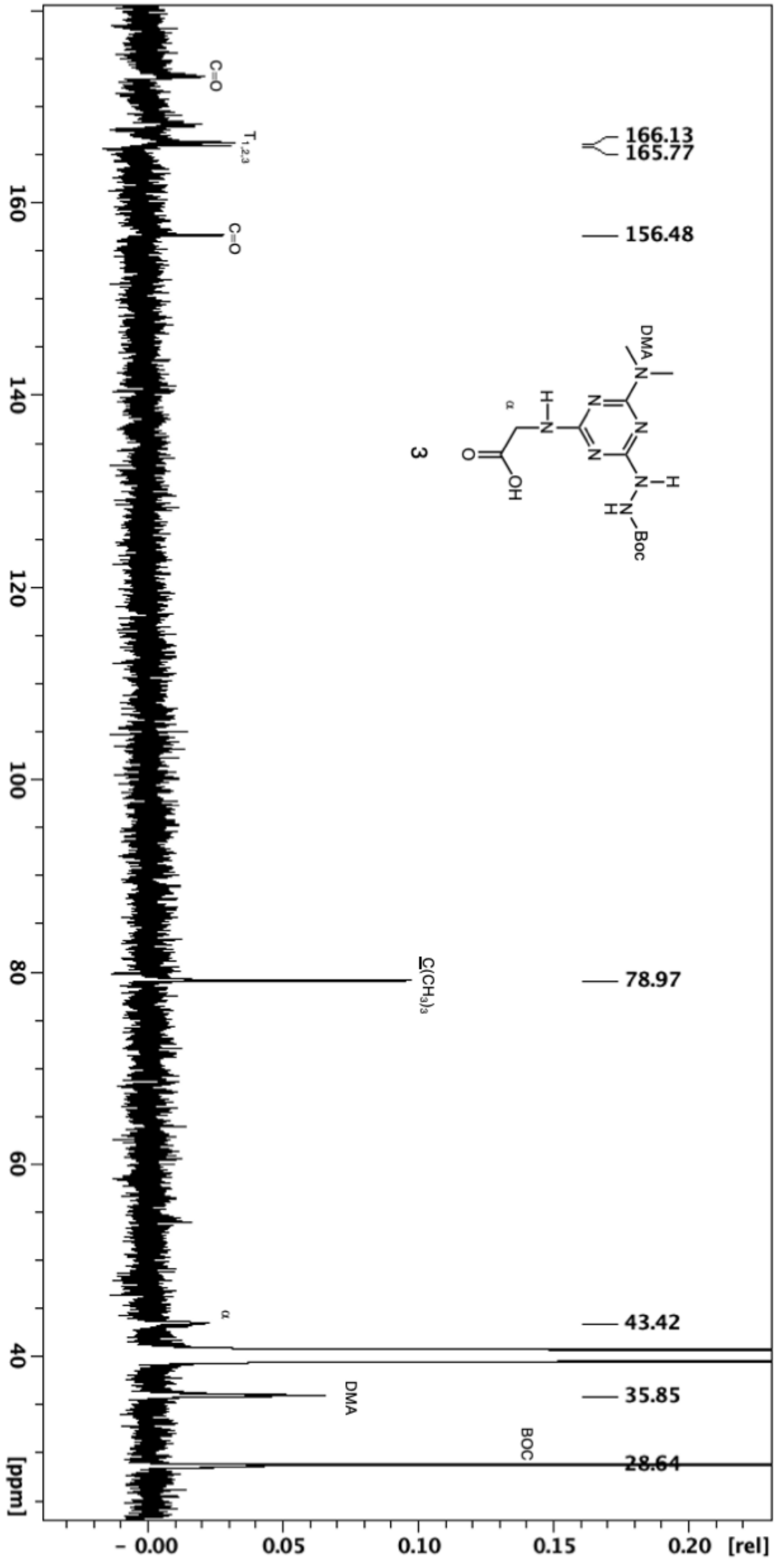

References

[1] Gaussian 16, Revision C.01, M. J. Frisch, G. W. Trucks, H. B. Schlegel, G. E. Scuseria, M. A. Robb, J. R. Cheeseman, G. Scalmani, V. Barone, G. A. Petersson, H. Nakatsuji, X. Li, M. Caricato, A. V. Marenich, J. Bloino, B. G. Janesko, R. Gomperts, B. Mennucci, H. P. Hratchian, J. V. Ortiz, A. F. Izmaylov, J. L. Sonnenberg, D. Williams-Young, F. Ding, F. Lipparini, F. Egidi, J. Goings, B. Peng, A. Petrone, T. Henderson, D. Ranasinghe, V. G. Zakrzewski, J. Gao, N. Rega, G. Zheng, W. Liang, M. Hada, M. Ehara, K. Toyota, R. Fukuda, J. Hasegawa, M. Ishida, T. Nakajima, Y. Honda, O. Kitao, H. Nakai, T. Vreven, K. Throssell, J. A. Montgomery, Jr., J. E. Peralta, F. Ogliaro, M. J. Bearpark, J. J. Heyd, E. N. Brothers, K. N. Kudin, V. N. Staroverov, T. A. Keith, R. Kobayashi, J. Normand, K. Raghavachari, A. P. Rendell, J. C. Burant, S. S. Iyengar, J. Tomasi, M. Cossi, J. M. Millam, M. Klene, C. Adamo, R. Cammi, J. W. Ochterski, R. L. Martin, K. Morokuma, O. Farkas, J. B. Foresman, and D. J. Fox, Gaussian, Inc., Wallingford CT, 2019.

[2] J.-D. Chai and M. Head-Gordon, "Long-range corrected hybrid density functionals with damped atom-atom dispersion corrections," Phys. Chem. Chem. Phys., 10 (2008) 6615-20. DOI: 10.1039/B810189B

[3] (a) R. Ditchfield, W. J. Hehre, and J. A. Pople, "Self-Consistent Molecular Orbital Methods. 9. Extended Gaussian-type basis for molecular-orbital studies of organic molecules," J. Chem. Phys., 54 (1971) 724. DOI: 10.1063/1.1674902; (b) W. J. Hehre, R. Ditchfield, and J. A. Pople, "Self-Consistent Molecular Orbital Methods. 12. Further extensions of Gaussian-type basis sets for use in molecular-orbital studies of organic-molecules," J. Chem. Phys., 56 (1972) 2257. DOI: 10.1063/1.1677527; (c) P. C. Hariharan and J. A. Pople, "Accuracy of AH equilibrium geometries by single determinant molecular-orbital theory," Mol. Phys., 27 (1974) 209-14. DOI: 10.1080/00268977400100171; (d) T. Clark, J. Chandrasekhar, G. W. Spitznagel, and P. v. R. Schleyer, "Efficient diffuse function-augmented basis-sets for anion calculations. 3. The 3-21+G basis set for 1st-row elements, Li-F," J. Comp. Chem., 4 (1983) 294-301. DOI: 10.1002/jcc.540040303; (e) M. J. Frisch, J. A. Pople, and J. S. Binkley, "Self-Consistent Molecular Orbital Methods. 25. Supplementary Functions for Gaussian Basis Sets," J. Chem. Phys., 80 (1984) 3265-69. DOI: 10.1063/1.447079.

[4] A. V. Marenich, C. J. Cramer, and D. G. Truhlar, "Universal solvation model based on solute electron density and a continuum model of the solvent defined by the bulk dielectric constant and atomic surface tensions," J. Phys. Chem. B, 113 (2009) 6378-96. DOI: 10.1021/jp810292n.

[5] J. J. P. Stewart, "Optimization of parameters for semiempirical methods. V. Modification of NDDO approximations and application to 70 elements," J. Mol. Model., 13 (2007) 1173-213. DOI: 10.1007/s00894-007-0233-4
